# Prostate Region-Wise Imaging Biomarker Profiles for Risk Stratification and Biochemical Recurrence Prediction

**DOI:** 10.3390/cancers15164163

**Published:** 2023-08-18

**Authors:** Ángel Sánchez Iglesias, Virginia Morillo Macías, Alfonso Picó Peris, Almudena Fuster-Matanzo, Anna Nogué Infante, Rodrigo Muelas Soria, Fuensanta Bellvís Bataller, Marcos Domingo Pomar, Carlos Casillas Meléndez, Raúl Yébana Huertas, Carlos Ferrer Albiach

**Affiliations:** 1 Radiation Oncology Department, Hospital Provincial de Castellón, 12002 Castellón, Spain; aalsanchezi@gmail.com (Á.S.I.); vmorill@gmail.com (V.M.M.); rodmuelas@hotmail.com (R.M.S.); 2 Quantitative Imaging Biomarkers in Medicine (Quibim), 46021 Valencia, Spain; alfonsopico@quibim.com (A.P.P.); almudenafuster@quibim.com (A.F.-M.); annanogue@quibim.com (A.N.I.); fuensantabellvis@quibim.com (F.B.B.); marcosdomingo@quibim.com (M.D.P.); raulyebana@quibim.com (R.Y.H.); 3 Radiodiagnosis Department, Hospital Vithas Castellón, 12004 Castellón, Spain; casillasmel@gmail.com

**Keywords:** prostate cancer, MRI, radiomics, imaging biomarkers, diffusion parameters, perfusion parameters, biochemical recurrence, risk

## Abstract

**Simple Summary:**

In prostate cancer (PCa), an accurate patient risk stratification, as well as the awareness of a possible biochemical recurrence (BCR) event, are crucial to individualize treatment decisions. Magnetic resonance imaging (MRI) is commonly used in the diagnosis, risk stratification, localization, and staging of PCa. Likewise, radiomics, which allows the extraction of quantitative parameters from medical images, has attracted increased attention in recent years. A combination of both strategies may be useful for predicting important clinical outcomes in these patients. In patients with localized PCa receiving neoadjuvant androgen deprivation therapy and radiotherapy, we explore the existence of putative prostate region-wise imaging biomarker (radiomic, diffusion, and/or perfusion features) profiles extracted from MRIs in order to discriminate patients according to their risk or the appearance of BCR 10 years after diagnosis, as well as to determine their predictive value alone or in combination with clinical variables.

**Abstract:**

Background: Identifying prostate cancer (PCa) patients with a worse prognosis and a higher risk of biochemical recurrence (BCR) is essential to guide treatment choices. Here, we aimed to identify possible imaging biomarker (perfusion/diffusion + radiomic features) profiles extracted from MRIs that were able to discriminate patients according to their risk or the occurrence of BCR 10 years after diagnosis, as well as to evaluate their predictive value with or without clinical data. Methods: Patients with localized PCa receiving neoadjuvant androgen deprivation therapy and radiotherapy were retrospectively evaluated. Imaging features were extracted from MRIs for each prostate region or for the whole gland. Univariate and multivariate analyses were conducted. Results: 128 patients (mean [range] age, 71 [50–83] years) were included. Prostate region-wise imaging biomarker profiles mainly composed of radiomic features allowed discriminating risk groups and patients experiencing BCR. Heterogeneity-related radiomic features were increased in patients with worse prognosis and with BCR. Overall, imaging biomarkers profiles retained good predictive ability (AUC values superior to 0.725 in most cases), which generally improved when clinical data were included (particularly evident for the prediction of the BCR, with AUC values ranging from 0.841 to 0.877 for combined models and sensitivity values above 0.960) and when models were built per prostate region vs. the whole gland. Conclusions: Prostate region-aware imaging profiles enable identification of patients with worse prognosis and with a higher risk of BCR, retaining higher predictive values when combined with clinical variables.

## 1. Introduction

Prostate cancer (PCa), one of the most commonly occurring urologic malignancies [1], is the second most common cancer in men worldwide and the second leading cause of cancer death in men [2], with an estimated 1,414,259 new cancer cases and 375,304 deaths in 2020, according to Globocan [3]. The worldwide PCa burden is estimated to continue increasing due to global population growth and aging, with 2.43 million new cases and 740,000 deaths by 2040 [4]. Although most PCa patients are diagnosed at an early stage, when curative treatment with surgery and/or radiation therapy can be attempted, a significant number of men who are treated with curative intent will fail primary treatment (27–53%) [5]. To overcome this variability, several risk stratification systems that aid in distinguishing aggressive indolent from aggressive tumors have been developed.

The National Comprehensive Cancer Network (NCCN) risk-stratification system has been widely used for many years, constituting the basis of treatment recommendations used for localized PCa throughout the world. Initially based on the risk classification proposed by D’Amico in 1998 [6], in which patients were divided into three different categories according to the prostate-specific antigen (PSA) level, Gleason score, and clinical T stage, the NCCN risk-stratification was updated in 2019 [7]. This new version includes a sub-classification of the intermediate-risk (IR) category into favorable-IR and unfavorable-IR groups, resulting in a total of five risk categories: Very low, low, intermediate (favorable or unfavorable), high, and very high [7]. NCCN risk groups have the primary intent to predict a patient’s pretreatment risk of biochemical recurrence (BCR) after curative treatment, as it has been associated with an increased risk of developing distant metastasis and dying of PCa [8]. Despite its wide adoption at present, NCCN risk stratification in clinical practice remains challenging, as it is not accurate enough [9]. Its suboptimal prognostic and discriminatory performance is evident for different patient groups, including those treated with radiotherapy (RT) and androgen deprivation therapy (ADT). Indeed, it has been demonstrated that ADT is known to cause significant changes in the appearance of the prostate on magnetic resonance imaging (MRI), which may lead to an underestimation of the tumor presence after treatment, hampering the assessment of treatment response [10,11]. Consequently, developing new tools for risk classification and BCR prediction is of utmost importance for PCa patients.

In recent years, radiomics has emerged as a promising tool in the field of medical imaging. Radiomics refers to a high-throughput quantitative imaging analysis method (also known as texture analysis) that extracts a large number of features from medical images [12]. These features allow characterizing the underlying tumor microarchitecture and heterogeneity, which is subtle and not immediately visible [13]. Indeed, radiomic features extracted from MRI have proven to be useful in PCa detection and localization [14,15,16,17,18], as well as in clinical outcome prediction [19]. Thus, texture features derived from apparent diffusion coefficient (ADC) and T2-weigthed (T2-w) MRI together with sample augmentation allowed obtaining a fairly accurate classification of Gleason patterns [14], while classifiers using features extracted from MRI performed well in identifying and localizing prostate cancer on MRI, discriminating between cancer and normal prostate tissue in the central gland and the Peripheral Zone (PZ) [15,18]. Finally, textural features also appear to be strongly associated with biochemical recurrence following PCa radiotherapy [19].

The aim of this study was to identify radiomic profiles in MRIs acquired after ADT initiation: (1) To identify patients with a worse prognosis (based on their stratification risk groups) and (2) to predict BCR 10 years after diagnosis. Additionally, an exploratory endpoint to identify radiomic profiles in patients with high/unfavorable-IR for BCR prediction was also conducted. All the analyses were performed in the whole prostate and per prostate region to additionally evaluate whether a region-wise analysis might influence our results.

## 2. Materials and Methods

### 2.1. Study Design and Patient Population

This was a retrospective single-center observational study conducted in accordance with the Declaration of Helsinki and approved by the Institutional Review Board of ethics committee from Castellón Provincial Hospital (Castellón de la Plana, Spain).

**Figure 1:**
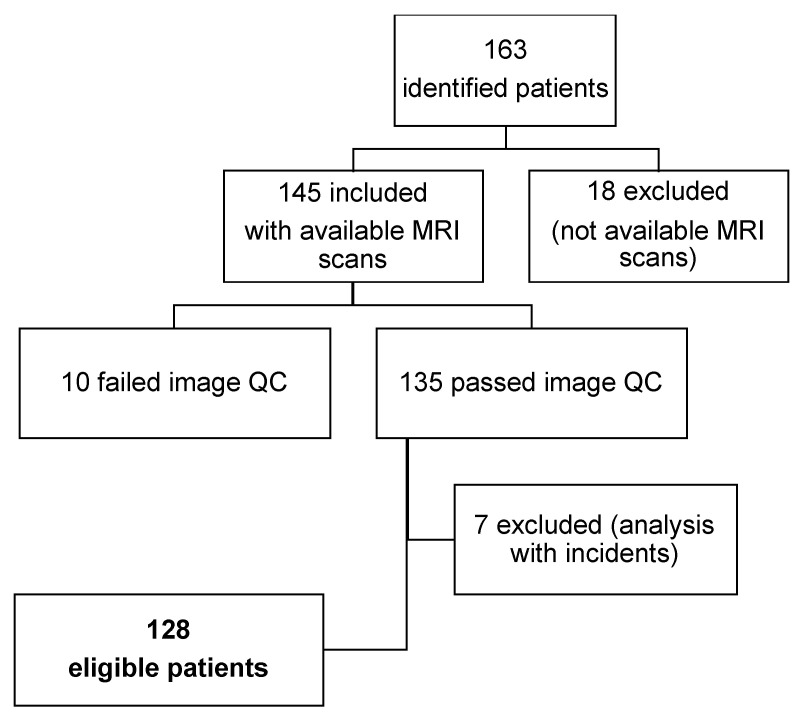
Flow chart of patient disposition. QC = quality check.

Patients meeting the following inclusion criteria were included: (1) Histological diagnosis of PCa, (2) treatment with neoadjuvant ADT, (3) locoregional treatment with external beam radiation therapy, (4) available baseline staging MRIs after hormonal treatment initiation, (5) assessment by analytical determination of PSA. Patients were excluded if they received previous RT for other reasons, if they had other neoformative processes at the pelvic level, or if MR images presented artifacts impeding their correct evaluation. A total of 163 patients were identified, of which 18 were excluded because medical images could not be recovered. For the remaining 145 cases, a quality check (QC) of their medical images was performed, which led to the exclusion of another 10 cases. Finally, incidents during image analysis were reported in 7 cases. As a result, a total of 128 patients met the eligibility criteria to be included in the study (Figure 1).

### 2.2. Risk Stratification and Biochemical Recurrence

Patients were classified according to the risk of BCR after definitive local therapy as proposed by NCCN guidelines v 2.2019, which uses a minimum of stage, grade, and PSA [7]. According to an adapted version of that risk stratification system, in this study, patients were categorized into three different risk groups: Low, intermediate (including favorable and unfavorable risk patients), and high. For a simplification of the analysis, risk groups were finally aggregated into two categories. Thus, group 1 included low-risk patients together with those with favorable-IR. Group 2 included high-risk patients grouped with unfavorable-IR patients.

BCR 10 years from diagnosis was defined according to the PHOENIX criteria as a rise by ≥2 ng/mL above the nadir PSA level [20]. Patients were classified based on the presence or absence of BCR 10 years after diagnosis.

### 2.3. MRI Acquisition

MRI exams included T2-w, dynamic contrast-enhanced (DCE), and diffusion-weighted imaging (DWI) sequences in axial orientation. Images were acquired in a Siemens 1.5 T MRI unit (Magnetom Avanto fit, Siemens Healthineers, Erlangen, Germany) once ADT treatment had been initiated, with the patient lying in a prone position.

For DCE series, high-spatial-resolution three-dimensional T1-weighted imaging was performed by using a 3D gradient-echo sequence (TE = 1.6 ms, TR = 4.69 ms, flip angle = 12°, isotropic voxel size of 1.6 mm, field of view of 100 mm). A total of 50 dynamics were acquired with a temporal resolution of 3 s. Total DCE sequence duration was 2 min 30 s, including preparation pulses. The intravenous contrast used for the DCE was Gadolinyum DTPA, dose 2 mL/kg. DWI images were acquired by an echo–planar imaging sequence (TE = 89 ms, TR = 4400 ms, pixel size of 1.7 mm, slice thickness of 5 mm, b-values of 0, 500, and 1000 s^2^/mm).

Images were exported in DICOM format from the PACS of the hospital using medical imaging universal connector (MIUC) software, installed from Quibim S.L. MIUC v.5.4.1 is a software developed by Quibim that allows communication between the web platform and the hospital environment. Once installed in the hospital, MIUC allowed Quibim’s platform to request the necessary images from the hospital’s PACS. Thus, the required images from the included patients in the study were stored in the Quibim platform, based in Microsoft Azure.

A QC was performed on all the exams received on the Quibim platform. The QC was performed by QUIBIM’s imaging analysis technicians certified for good clinical practice (GCP) and with more than 5 years of experience in multi-modality image acquisition and QC in observational studies and clinical trials. The QC criteria were defined by a board-certified radiologist with more than 20 years of experience. For each imaging exam, the following aspects were reviewed:

- MRI sequences necessary for the subsequent analysis were present and complete (T2w and DWI or T2w, DWI, and DCE)
- Optimal contrast to noise ratio was verified on the whole sequences
- Full coverage within the field of view (FOV) and centered on the prostate
- Absence of artifacts that may affect the analysis (i.e., patient movement)

### 2.4. Automated Segmentation

Automatic prostate segmentation was performed by using QP-Prostate algorithms v. 1.0.6 (Quibim, S.L., Valencia, Spain). The QP-Prostate artificial intelligence algorithm, based on convolutional neuronal networks (CNNs), automatically detects prostate anatomy to identify and segment each prostate region, as well as other areas defined in the PI-RADS v2.1 guidelines [21]. Thus, an automated regional organ prostate segmentation, including the Transitional + Central Zone (TZ + CZ), the PZ, and the seminal vesicles, was performed (Supplementary Figure S1). The segmentation was automatically generated in the T2w sequence, and a registration of the DWI and the DCE to the T2w sequence was performed, allowing to extract diffusion and perfusion biomarkers, respectively.

### 2.5. Imaging Biomarkers

The analysis of imaging biomarkers included the evaluation of texture/radiomic features and quantitative parameters (diffusion and perfusion features) extracted from T2-w and DWI and DCE sequences, respectively.

#### 2.5.1. Texture Analysis

These imaging biomarkers provide information on tissue heterogeneity related to tumor progression. A total of 105 radiomic features were extracted from each region that can be classified into different categories:

1. Shape features: The quantitative description of the region of interest (ROI)s’ geometric properties, such as surface area, total volume, diameter, elongation, sphericity, and surface-to-volume ratio.
2. First-order statistics (histogram-based features): These describe the distribution of voxel intensities within the image ROI through commonly used conventional metrics (e.g., energy, entropy, mean, interquartile range, skewness, kurtosis, and uniformity).
3. Second-order statistics (textural features): These are obtained from secondary matrices that include statistical inter-relationships between neighboring voxels, such as:
  - Gray-level Co-occurrence Matrix (GLCM): The spatial distribution of gray-level intensities within a 3D image.
  - Gray-level Run-length Matrix (GLRLM): The number of contiguous voxels that have the same gray-level value. This characterizes the gray-level run lengths of different gray-level intensities in any direction.
  - Gray-level Size-zone Matrix (GLSZM): This quantifies gray-level zones, i.e., the number of connected voxels that share the same gray-level intensity, in a 3D image.
  - Neighboring Gray-Tone Difference Matrix (NGTMD): This quantifies the difference between a gray value and the average gray value of its neighbors within a distance δ.
  - Gray-level Dependence Matrix (GLDM): This quantifies the number of connected voxels within a distance δ that are dependent on the center voxel.

#### 2.5.2. Diffusion Parameters

Diffusion parameters were obtained through QP-Prostate (Quibim, S.L). DWI-MR sequence enabled the evaluation of water molecule behavior in tissues. Diffusion imaging biomarkers were quantified using the Gaussian mono-exponential mathematical model. For each ROI, 5 parameters, including the standard deviation (std) and percentiles 25, and 75 of the ADC were computed: *ADC_mean*, *ADC_std*, *ADC_median*, *ADC_p25*, and *ADC_p75*.

The ADC is a measurement of the magnitude of the random motion of water molecules within tissue, and its corresponding parametric map reflects the degree of diffusion in the region under study and provides the ADC value in units of mm^2^/s. ADC values < 1.0 to 1.1 × 10^−3^ are generally acknowledged in adults as indicating restriction. However, it is entirely dependent on the organ being imaged and the pathology [22].

#### 2.5.3. Perfusion Parameters

Perfusion parameters were obtained through QP-Prostate (Quibim, S.L). The DCE perfusion quantifies perfusion parameters evaluating T1 shortening effects induced by the gadolinium-based contrast bolus passing through the tissue. Regional increased signal is due to gadolinium concentration which will depend on different factors. A one-input two-compartment pharmacokinetic model will also be applied to obtain the transfer constant (K^trans^), the extravasation constant (k_ep_), and the interstitial volume fraction (v_e_), according to the simplified Tofts model [23,24]:

$$C_{t}(t)=K^{trans}\int_{0}^{t}C_{AIF}\left(\tau \right)e^{-k_{ep}*\left(t-\tau \right)}\cdot d\tau \;v_{e}=\frac{K^{trans}}{k_{ep}}$$

Median, standard deviation (std) and percentiles of K^trans^, k_ep_, and v_e_ were extracted from each ROI:

- *K^trans^_mean*, *K^trans^_Std*, *K^trans^_median*, *K^trans^_p25*, *K^trans^_p75*
- *k_ep__mean*, *k_ep__Std*, *k_ep__median*, *k_ep__p25*, *K_ep__p75*
- *ve_mean*, *ve_std*, *ve_median*, *ve_p25*, *Ve_p75*

Perfusion parameters are dependent on the arterial input function (AIF), which was automatically determined and whose accuracy was manually reviewed by image technicians from Quibim and modified if necessary.

#### 2.5.4. Weighted Biomarkers Averaging for the Whole Prostate

Given that the imaging biomarkers were extracted from each region (CZ + TZ, PZ, and seminal vesicles), a weighted average considering each region volume was calculated for the whole prostate. The weighted average was not performed in shape features. As previously described by Chang et al. [25], shape features from the three regions were aggregated, while a weighted average according to the volume of each prostate region was calculated for the other imaging biomarkers (1st-order and 2nd-order texture features, diffusion, and perfusion parameters). The following formulas were applied:

$$ROI\;weight=\frac{volume\;of\;the\;ROI}{\begin{array}{c} \mathrm{sum}\;\mathrm{of}\;\mathrm{the}\;\mathrm{volumes}\;\mathrm{of}\;\mathrm{the}\;\mathrm{three}\;\mathrm{ROIs} \end{array}}$$

$$Weighted\;average\;F1=\left(ROI1weight\;*\;F1\right)+\left(ROI2weight\;*\;F1\right)+\left(ROI3weight\;*\;F1\right)$$

where F1 is an extracted feature from the 1st- or 2nd-order texture, diffusion, or perfusion analysis.

### 2.6. Statistical Analysis

#### 2.6.1. Univariate Analysis

For each endpoint, a correlation matrix was computed to eliminate highly correlated variables. Quantitative variables with a Pearson’s coefficient greater than 0.9 were discarded. Prior to the evaluation of the relationship between radiomic features and clinical endpoints, Shapiro–Wilk’s test and Levene’s test were run to assess normality and homoscedasticity, respectively. In the case of normal distributions, comparisons between groups were performed applying a Student’s *t*-test, while the Wilcoxon rank sum test was used for variables with non-normal distributions. A *p*-value less than 0.05 was considered statistically significant.

#### 2.6.2. Multivariate Analysis

Different predictive models were developed to assess the ability of imaging features, alone or in combination with clinical variables, to predict risk groups and BCR in the whole population and in the high/unfavorable-IR group (exploratory endpoint). For this purpose, multiple GLM’s (logit link function) were computed using the variables that resulted to be statistically significant in the univariate analysis performed for each prostate region and for the whole prostate, with a 5-fold cross-validation as the optimization method. Any independent variable with a variance inflation factor (VIF) larger than 5 was eliminated to prevent multicollinearity. The smallest Akaike information criterion (AIC) was used to determine the most parsimonious combinations of variables in each case. The final models were the result of a backward variable selection process, choosing the most parsimonious model in each case due to its low AIC. For each endpoint and each prostate region, as well as for the whole prostate, a predictive model including imaging features only or a combination of both imaging and clinical variables was built. For the risk group prediction in the total population and in the high/unfavorable-IR population (exploratory endpoint), the following variables obtained at diagnosis were included in the combined models: Eastern cooperative oncology group performance status (ECOG PS), age, and perineural invasion obtained from the biopsy. For the BCR prediction, in addition to ECOG PS, age, and perineural invasion, the following ones were also included: PSA, The International Society of Urological Pathology (ISUP) grade, and node status (N) from TNM stage.

All the analyses were performed using R v.4.2.2 and RStudio v.2022.07.2+576 software.

## 3. Results

### 3.1. Clinical Characteristics

Out of the total 128 patients included, T2w sequences were available in all of them, while DWI and DCE sequences were available in 107 and 62 patients, respectively. The mean age (range) of the 128 patients was 71 (50–83) years and the mean (range) PSA level was 20.90 (1–627) ng/mL. Main patient characteristics are summarized in Table 1.

### 3.2. Imaging Biomarker Profiles to Define Patient Stratification Risk

**Figure 2:**
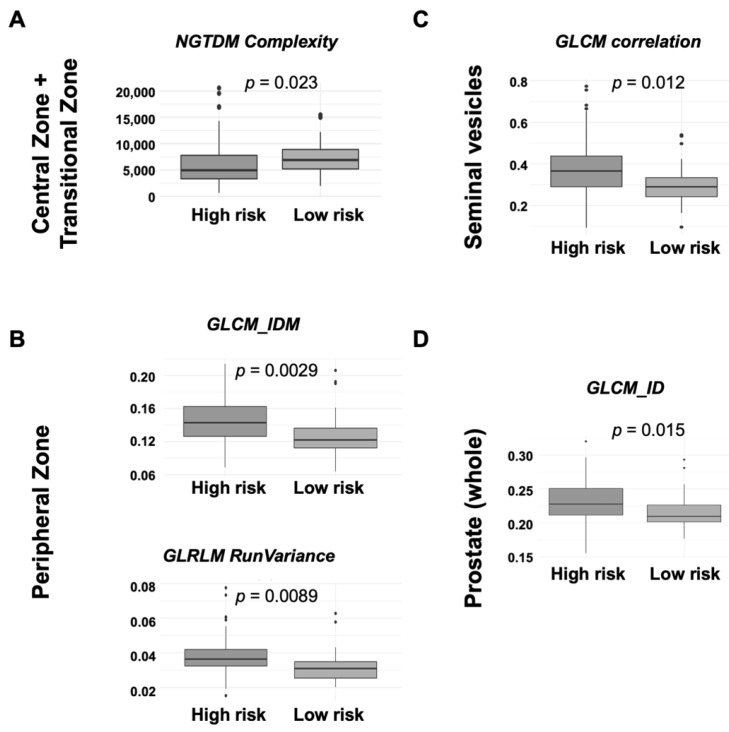
Box plots for imaging (radiomics) biomarkers in each prostate region for risk stratification (arbitrary units). (**A**) Central Zone + Transitional Zone; (**B**) Peripheral Zone; (**C**) Seminal vesicles; (**D**) Prostate (whole). GLCM = Gray-Level Co-Occurrence Matrix; GLRLM = Gray Level Run Length Matrix; ID = inverse difference; IDM = inverse different moment.

Patient distribution according to the available MRI sequences and their stratification risk is detailed in Table S1. The analysis conducted in the different prostate regions, as well as in the whole prostate, revealed statistically significant differences between high/unfavorable-IR and low/favorable-IR groups in terms of radiomic features (Figure 2). No changes were detected for diffusion and perfusion parameters.

#### 3.2.1. Central Zone and Transitional Zone

In this subregion, values for the *NGTDM_complexity* feature, which measures the non-uniformity and rapid changes in gray levels [26], resulted to be significantly higher (*p* = 0.023) in low/favorable-IR patients, although the number of outliers was high in this analysis (Figure 2A).

#### 3.2.2. Peripheral Zone

Two radiomic features, *GLCM_Inverse Difference Moment* and *GLRLM_Run Variance*, both related to texture heterogeneity, presented statistically significant lower values in patients with low/favorable-IR (*p* = 0.0029 and *p* = 0.0089, respectively) in the PZ (Figure 2B).

#### 3.2.3. Seminal Vesicles

In the seminal vesicles, the only radiomic feature that allowed distinguishing patients according to their risk was the *GLCM_Correlation* feature, with a lower value (*p* = 0.0012) found in low/favorable-IR patients, although the number of outliers was high in this analysis (Figure 2C).

#### 3.2.4. Whole Prostate Gland

A radiomic feature measuring the contrast between the gray levels of contiguous pixels, *GLCM_Inverse Difference*, and, therefore, indicative of a more heterogeneous or complex texture, resulted to be significantly increased (*p* = 0.015) in high/unfavorable-IR patients (Figure 2D).

### 3.3. Imaging Biomarker Profiles to Define Biochemical Relapse

Patient distribution according to the available MRI sequences and the presence or absence of BCR 10 years from diagnosis is detailed in Table S2. As summarized in Table 2 and in Supplementary Figures S2–S4, in all prostate regions, as well as in the whole gland, different radiomic features and one diffusion biomarker (only for CZ + TZ) allowed differentiating patients suffering BCR from those who did not.

#### 3.3.1. Central Zone and Transitional Zone

In the CZ + TZ, *GLRLM_Short Run Emphasis* was significantly lower (*p* = 0.0311) in patients presenting BCR, a variable that measures the distribution of short run lengths and for which greater values may be indicative of more fine textural features [26]. In these patients, however, another radiomic feature, *GLSZM_Large Area Emphasis*, measuring the distribution of large area size zones, and for which greater values indicate larger size zones and more coarse textures resulted to be significantly higher (*p* = 0.0249) (Table 2 and Supplementary Figure S2A). Therefore, CZ + TZ is characterized by less fine but more coarse texture features in patients with BCR 10 years after the diagnosis.

Furthermore, this was the only subregion in which a diffusion biomarker allowed discriminating between the two groups. Thus, *ADC_mean* was significantly lower (*p* = 0.0292) in patients experiencing BCR (Table 2 and Supplementary Figure S2A).

#### 3.3.2. Peripheral Zone

In this subregion, three shape radiomic features allowed distinguishing patients with or without BCR. Values for two of them, *Major Axis Length* and *Maximum 3D Diameter*, were significantly lower (*p* = 0.0019 and *p* = 0.0236, respectively) in patients with BCR, while in the remaining one, *Flatness*, an increase compared to patients not experiencing BCR was detected (*p* = 0.0301) (Table 2 and Supplementary Figure S2B). As observed, a larger diameter and a greater axis length of the PZ were characteristic of patients not experiencing BCR.

#### 3.3.3. Seminal Vesicles

In the seminal vesicles, up to 16 radiomic features were significantly different in patients experiencing BCR after 10 years from diagnosis compared to those who did not relapse (Table 2 and Supplementary Figure S3). These variables included first-order, shape, and high-order features. Interestingly, some of them, directly related to texture heterogeneity, such as *Skewness* and *NGTDM_Strength*, were significantly increased in the group of patients with BCR (*p* = 0.0036 and *p* = 0.0009, respectively). Additionally, in this group, two texture complexity-related radiomic features were also significantly increased and decreased, respectively (*GLCM_ Informational Measure of Correlation1* and *GLCM_ Informational Measure of Correlation2*; *p* = 0.0079 and *p* = 0.0334, respectively).

#### 3.3.4. Whole Prostate Gland

In the whole prostate (Table 2 and Supplementary Figure S4), as observed in other prostate subregions, radiomic features related with a higher heterogeneity, such as *Skewness* and *GLCM_Inverse Difference*, characterized the group of patients presenting BCR 10 years after diagnosis (*p* = 0.0018 and *p* = 0.0079, respectively). Moreover, as in the PZ, *Major Axis Length* was significantly lower (*p* = 0.0057) in patients with BCR.

### 3.4. Imaging Biomarker Profiles to Define Biochemical Relapse in High/Unfavorable-Intermediate Risk Patients, Exploratory Analysis

Table S3 summarizes patient distribution according to the available MRI sequences and the presence or absence of BCR 10 years from diagnosis in this patient subgroup. In all prostate regions, except for the PZ, several radiomic features were found to be significantly different between patients experiencing BCR 10 years after diagnosis vs. those who did not among patients with high/unfavorable-IR (Supplementary Figures S5 and S6). Moreover, ADC-related parameters were significantly different in these two groups in all prostate subregions except for CZ + TZ. As for the other endpoints, no changes were detected in perfusion parameters.

#### 3.4.1. Central Zone and Transitional Zone

This subregion was characterized by changes in heterogeneity-related radiomic features, such as *GLSZM_Large Area Emphasis*, were was significantly higher (*p* = 0.0145) in patients with BCR (Supplementary Figure S5A).

#### 3.4.2. Peripheral Zone

A lower value (*p* = 0.0459) of the *ADC_percentile 25* was observed in patients experiencing BCR (Supplementary Figure S5B).

#### 3.4.3. Seminal Vesicles

In the seminal vesicles, six radiomic features and one diffusion biomarker were significantly different between the two groups. In patients with high/unfavorable-IR, *Major Axis Length* and *Maximum 2D Diameter Column* were significantly lower (*p* = 0.0239 and *p* = 0.0077, respectively). Likewise, *ADC_percentile 75* was decreased in these patients (*p* = 0.008) (Supplementary Figure S6).

#### 3.4.4. Whole Prostate Gland

Among the nine radiomic features with significant differences between both groups, *Skewness* and *GLSZM Large Area Emphasis* were increased in relapsing patients (*p* = 0.0327 and *p* = 0.0149, respectively), suggesting a higher heterogeneity and more coarse textures. As in specific prostate regions, an ADC parameter, *ADC_mean*, was observed to be decreased in patients relapsing 10 years after diagnosis (Supplementary Figure S7).

### 3.5. Predictive Models

Given that imaging biomarker profiles were able to discriminate patients according to their risk and the appearance of BCR 10 years after diagnosis, we developed several logistic regression models to evaluate whether those imaging biomarkers retained predictive ability for each of the endpoints considered. A summary of the results is provided in Table 3.

Overall, models showed good performance, with AUC values superior to 0.725 in most of the cases. For the two co-primary endpoints, only some imaging models analyzing specific prostate regions and not those developed for the whole prostate resulted to be statistically significant. When including clinical variables, a general improvement in model performance was observed, again with statistically significant results only for models developed for specific prostate regions. This improvement achieved by imaging and clinical models compared to imaging models was particularly evident for the prediction of the BCR, with AUC values ranging from 0.841 to 0.877 for combined models. Remarkably, models including imaging features extracted from the PZ, alone or in combination with clinical variables, allowed significantly predicting risk groups and BCR. Thus, for the prediction of risk groups, the imaging features-based model yielded an AUC value of 0.670, with an accuracy of 0.648, a sensitivity of 0.667, and a specificity of 0.631 (*p* = 0.025), while for the appearance of BCR 10 years after diagnosis, both the imaging and the imaging + clinical variables models achieved significant results, with AUC, accuracy, sensitivity and specificity values of 0.748, 0.822, 0.977, and 0.150 (*p* = 0.025), and 0.877, 0.860, 0.294, and 0.976 (*p* = 0.002), respectively.

In terms of the exploratory analysis, a combination of both imaging features and clinical variables allowed significantly predicting BCR 10 years after diagnosis in high/unfavorable-IR patients in all the prostate regions analyzed, as well as in the whole prostate gland, although with low specificity values.

## 4. Discussion

PCa is characterized by its significant heterogeneity, which implies a wide range of oncologic prognoses [27,28]. The majority of prostate cancers are slow-growing, but the remaining cases can be extremely aggressive and even fatal [29,30]. Therefore, it is crucial to build highly discriminative prognostic models that enable classifying patients based on their risk, allowing more accurate therapeutic decisions. Likewise, the pretreatment identification of those patients with localized PCa who will experience BCR can be relevant to guide treatment decisions and retains an important prognosis value as well, especially for high-risk patients. Regrettably, current stratification tools have important limitations and do not allow an accurate classification of certain patient subgroups [9]. In this study, we have tested the applicability of MRI-derived biomarkers for the identification of patients with worse prognosis and a higher risk of BCR relapse 10 years after diagnosis. Thus, our results demonstrate that patients from different risk groups (low/favorable-IR vs. high/unfavorable-IR) are characterized by specific imaging profiles (mainly composed of radiomic features), which are different depending on the prostate region. In the same way, specific region-aware imaging profiles also allowed us to discriminate between patients experiencing or not BCR 10 years after diagnosis, both in the whole population and specifically in patients with high/unfavorable-IR. Logistic regression models developed with imaging features alone or in combination with clinical variables confirmed the predictive value of imaging biomarkers and the relevance of our prostate region-wise analysis. These results not only highlight the potential of a non-invasive method based on images that are usually collected during routine clinical care and, consequently, could be easily implemented but are also especially relevant for patients treated with ADT, such as those analyzed here. Thus, in our study, these patients had already started the therapy, a scenario in which conventional stratification tools may lead to an underestimation of risk as a result of the effects of therapy. Our approach offers a novel alternative to improve prediction and risk estimations in patients receiving ADT prior to MRI acquisition.

Overall, in different prostate regions, increased values of texture heterogeneity-related features were observed in the groups with worse outcomes. Thus, radiomic features such as *GLCM_Inverse Difference Moment, GLRLM_Run Variance, GLCM_Inverse Difference*, *Skewness* and *NGTDM_Strength* showed higher values in high/unfavorable-IR patients compared to those with a lower risk, and in those experiencing BCR 10 years after diagnosis compared to those who did not. Furthermore, texture complexity-related variables, and features associated with more coarse textures, such as *GLCM_ Informational Measure of Correlation1* and *GLCM_ Informational Measure of Correlation2* and *GLSZM_Large Area Emphasis*, were also increased in patients with relapsing disease in different prostate regions. These findings are in agreement with the widely accepted hypothesis that links the concept of texture heterogeneity, at least at the tumor level, with poorer prognosis, which could be secondary to intrinsic aggressive biology or treatment resistance [31], and that has been observed in different oncological diseases such as breast cancer, lung cancer or PCa [31]. Another relevant finding provided by radiomic features was related to prostate size and prognosis. Thus, at least for the prediction of BCR in both the total population and the high/unfavorable-IR group, a significant decrease in features measuring the gland, such as *Major Axis Length, Maximum 2D Diameter*, or *Maximum 3D Diameter*, was observed in patients experiencing relapse in different prostate regions including the PZ and seminal vesicles, as well as in the whole prostate. These results are in agreement with different studies that support the hypothesis that a large prostate size may be protective against PCa when compared to smaller prostates [32] and with certain results which indicate that PCa patients with large prostates have a better prognosis [33,34]. It is important to highlight that some discrepancies were also found in some other radiomic features. For example, *NGTDM_complexity* in the CZ + TZ or *GLCM_Correlation* in the seminal vesicles for patient risk stratification results were contrary to expected. However, the elevated number of outliers in these and other similar cases might be explaining these outcomes. Other important imaging biomarkers, such as diffusion and perfusion parameters, have also been related with tumor heterogeneity [35,36,37]. In this study, although no perfusion parameters were able to discriminate between the different analyzed groups, diffusion variables allowed classifying patients with or without BCR, both in the whole population and among patients with high/unfavorable-IR. Thus, in the CZ + TZ, *ADC_mean* was significantly lower in patients experiencing BCR 10 years after diagnosis in the whole population, while *ADC_percentile 25*, *ADC_percentile 75*, and *ADC_mean* were decreased in the PZ, seminal vesicles, and the whole prostate, respectively, in high/unfavorable-IR patients with BCR. It is well-known that, in general, tumors have lower ADC values, whereas normal/benign/reactive tissues have correspondingly higher values, as DWI MRI depends on the microscopic mobility of water, and most tumors have increased cellular density that restricts water mobility [37]. As a result, it is reasonable to think that in our patient cohort, prostate regions reflecting higher cellularity are indicative of a worse prognosis. Indeed, prior studies focusing on ADC analysis to predict prostate lesion risk consistently have identified *ADC_min*, *ADC_mean*, as well as *ADC_median*, as highly predictive (AUC range 0.72–0.90) [14,38].

Given that our results pointed out the existence of different region-wise imaging biomarker profiles allowing to discriminate patients according to their risk and the appearance of BCR 10 years after diagnosis, we decided to further explore the predictive ability of those features. The development of several logistic regression models demonstrated that diffusion and radiomic parameters (especially radiomic features as most of the profiles were mainly composed of these features) retain a significant predictive value. In particular, a general improvement in model performance was observed when clinical variables were included, with increased AUC values and *p*-values closer to the statistical significance. This was particularly evident in the prediction of the BCR both in the total population and in high/unfavorable-IR patients. A reason explaining these results would be related to the selected clinical variables included in the models. Thus, while for BCR prediction ECOG PS, age, perineural invasion, PSA, ISUP grade, and N from TNM stage were considered, for the risk stratification endpoint, only ECOG PS, age, and perineural invasion were included, as the other variables are implicit in the classification of risk groups as defined by the NCCN [7]. We hypothesize that ECOG PS, age, and perineural invasion are unlikely to be as informative as PSA, ISUP grade, or TNM stage, and consequently, do not provide an extra added value when included in the models. Importantly, in the high/unfavorable-IR population, all combined models (including imaging features and clinical variables) seemed to significantly outperform imaging models. Although these findings are of special interest to the patients for whom treatment strategies may be essential to improve their outcomes and prognosis, they must be interpreted cautiously, as further validations are required in greater and more balanced patient cohorts.

Another important conclusion that can be drawn from our results is related to our approach to the evaluation of the imaging biomarker profiles according to the different prostate regions. Thus, at least for the two co-primary endpoints, an overall improvement in model performance with statistically significant results was observed for models analyzing specific prostate regions compared to those analyzing the whole prostate. This stresses the relevance of performing region-aware analysis, especially for outcomes prediction. Our results are in line with those published by [39], who evaluated whether radiomic features for PCa detection from MRI in the TZ were similar to the features that are useful for PCa detection in the PZ. The authors found notable differences between the two regions and determined that a logistic regression classifier developed with PZ radiomic features was able to detect PZ tumors on an independent test set with significantly higher accuracy (AUC = 0.61–0.71) than a zone-ignorant classifier trained to detect cancer throughout the entire prostate. As a result, they concluded that decision support tools for evaluating prostate MRI exams should consider differences between TZ and PZ tumors. Interestingly, in our study, models including imaging biomarkers—mainly radiomic features—extracted from the PZ, alone or in combination with clinical variables, also showed good prediction ability with some of the highest AUC values among the analyzed models, allowing significantly predicting both risk groups and BCR. A possible explanation for this behavior in the PZ could lie in the fact that prostate tumors primarily arise from this region [40].

To the best of our knowledge, this is the first study demonstrating that region-wise imaging biomarker profiles mainly developed with imaging features extracted from MRIs in patients treated with RT and ADT are useful for predicting risk groups and BCR. The capability of texture features to make clinical predictions has been widely demonstrated in different clinical settings in PCa. For example, in 2018, Shiradkar et al. [13] demonstrated that radiomic features from pretreatment MRI could be predictive of PCa BCR after therapy (radical prostatectomy or RT) and might help identify men who would benefit from adjuvant therapy. This study was mainly based on Haralick features, a result of various statistics computed over a matrix of spatial voxel-intensity relationships that capture spatial intensity-based heterogeneity [41]. A similar approach with similar results associating Haralick features from the PZ with BCR was described by Gnep et al. [19]. In terms of risk stratification, the utility of radiomics has been demonstrated in several studies in which machine learning models were able to accurately assess PCa risk [42,43,44].

The landscape of biomarkers in PCa is rapidly evolving. Although PSA has been the go-to test for screening and diagnosis since 1986 [45], its use is becoming increasingly controversial, as it has been demonstrated to lead to overdiagnosis and overtreatment of PCa that is clinically insignificant [46,47]. Consequently, there is a clinical unmet need to develop biomarkers that can assist not only in screening stages to reduce unnecessary biopsies but also in disease management and monitoring. At present, molecular biomarker-based tests are numerous, but only a few of them are available for clinical use [46,47]. Existing and emerging assays can be classified into three categories: Tests for biopsy-naïve men (e.g., Prostate Health Index, Mi Prostate Score, 4K Score), tests for men with prior negative biopsies (e.g., ConfirmMDx, Progensa PCA3), and men on active surveillance (e.g., OncotypeDx, Prolaris, Decipher) [48]. Additionally, novel molecular biomarkers are continuously being discovered (e.g., PACE4-altCT, a new isoform of proprotein convertase PACE4 [49]), and the use of advanced technologies, such as nanotechnology—with the potential to alleviate the current limitations of molecular testing—[46], or data-driven deep learning approaches—known as cross-cancer learning—[50], is gaining attention in recent years. In this scenario, multiparametric MRI (mpMRI), considered by some experts as another “biomarker” for PCa, cannot be forgotten. This imaging modality, recommended by clinical guidelines as a primary screening system [8,51], is emerging as a tool to complement and enhance molecular biomarker testing, representing a promising technology for PCa screening, localization, staging, and risk stratification [46]. In comparison to other imaging tests, mpMRI of the prostate offers a higher soft-tissue resolution and multiple imaging data parameters non-invasively. This enables a better understanding of the entire prostate gland and its relationship to the environment, as well as better PCa staging guidance [52,53]. Quantitative parameters extracted from the different MRI sequences, such as ADC from DW-MRI, pharmacokinetic parameters, such as K^trans^, from DCE-MRI or radiomic features, are increasingly being utilized in multiple clinical studies to create predictive models for precise diagnosis and staging, therapy planning, post-treatment monitoring, and response prediction of PCa [54]. However, considering the available evidence, it is unlikely that a single biomarker achieves the required sensitivity and specificity to enable accurate predictions, and consequently, to be implemented in routine clinical practice. Combined strategies, such as those provided by MRI-derived imaging biomarkers, including radiomics, and molecular biomarkers, might be crucial in this setting. Finally, it is worth noting that, in the near future, mpMRI might coexist with new alternatives that are gaining great popularity in recent years, such as prostate-specific membrane antigen positron emission tomography/CT (PSMA PET/CT), multiparametric ultrasound (mpUS), whole-body MRI (WB-MRI), PSMA PET/MRI or micro-ultrasound (MUS) [55]. Although most of them are still supported by limited evidence, some of them might become relevant in the PCa field. Nevertheless, it is important to highlight that radiomics can be applied to various imaging techniques such as CT, MRI, PET, X-ray, and ultrasonography [56], making it reasonable to think that their application in these new imaging modalities, which combine and/or arise from classical imaging techniques, could also be possible.

This study also has some limitations. Firstly, it was a single-center study, and its application to data/patients from other institutions should be further explored. Secondly, the information on the primary tumor location could not be retrieved, making it difficult to interpret some of our results. Thirdly, we are aware that our predictive models for BCR were limited by their low specificity. Although this is undoubtedly a major drawback, it is important to note that our standard of reference were patients experiencing BCR 10 years after diagnosis. This means that a low specificity would include more false positive cases than desirable, but at least the high sensitivity values of those models would ensure an accurate prediction of the true positive cases, that is, of patients with BCR. Finally, although results for the exploratory endpoint were promising, it is important to highlight the notable unbalance of the high/unfavorable-IR patient cohort, which clearly limits the impact of our results. Indeed, this was a limitation also found in the overall cohort, as the groups considered for both co-primary endpoints were also unbalanced (low/favorable-intermediate risk patients, *n* = 32 vs. high/unfavorable-intermediate risk unfavorable group, *n* = 96 and patients experiencing biochemical relapse 10 years after diagnosis, *n* = 20 vs. patients who did not relapse, *n* = 108). Further studies with greater and more homogeneous populations are required. Despite these limitations, this work provides valuable insights about the importance of imaging biomarkers, especially radiomic features, for the prediction of important PCa outcomes in a set of patients for whom rapid and accurate decision-making processes are essential to ensure the best possible outcomes and prognosis.

## 5. Conclusions

Different prostate region-wise imaging biomarker profiles, mainly composed of radiomic features, were able to discriminate patients according to their risk or to the appearance of BCR 10 years after diagnosis. Furthermore, those imaging profiles, alone or in combination with clinical variables, allowed developing models to predict patient prognosis at risk level as well as BCR, both in the total population and specifically in high/unfavorable-IR patients, with good performance. This paves the way for new individualized patient management strategies, in which anticipating patient risk and prognosis may be essential to change the course of localized PCa, for example, by intensifying treatment in high-risk patients or by de-escalating it in those at low risk. These personalized strategies would reduce toxicity and positively impact patient’s quality of life. Further studies on bigger and more balanced patient cohorts are required to validate our results.

## Figures and Tables

**Table 1:** Patient characteristics at baseline, risk stratification and biochemical relapse.

Characteristic	Patients (*N* = 128) *n* (%)
ECOG PS	
0	2 (1.56)
1	73 (57.03)
2	53 (41.41)
ISUP	
1	38 (29.69)
2	34 (26.56)
3	23 (17.97)
4	16 (12.50)
5	17 (13.28)
c(N) from TNM stage	
cN0	113
cN1	15
Perineural invasion	
No	114 (89.06)
Yes	10 (7.81)
Unknown	4 (3.13)
Risk stratification	
Low	6 (4.69)
Favorable intermediate	26 (20.31)
Unfavorable intermediate	31 (24.22)
High	65 (50.78)
Biochemical relapse (10 years from diagnosis)	
Yes	20 (15.63)
No	108 (84.38)

ECOG PS = Eastern cooperative oncology group performance status; ISUP = The International Society of Urological Pathology.

**Table 2:** Statistically significant differences (*p*-values) in imaging biomarkers to classify patients according to the presence or absence of biochemical relapse 10 years after the diagnosis.

	Type	CZ + TZ	PZ	Seminal Vesicles	Whole Prostate
Texture analysis					
*10percentile*	First-order	–	–	0.0161	–
*Median*	First-order	–	–	0.0031	–
*Skewness*	First-order	–	–	0.0036	0.0118
*Flatness*	Shape 2D	–	0.030	–	–
*Major Axis Length*	Shape 2D	–	0.002	0.0159	0.0057
*Minor Axis Length*	Shape 2D	–	–	0.0261	
*Maximum 2D Diameter*	Shape 2D	–	–	0.0031	
*Maximum 3D Diameter*	Shape 3D	–	0.024	–	–
*Surface Volume Ratio*	Shape 3D	–	–	0.0141	–
*GLCM_Inverse Difference*	Second-order	–	–	–	0.0079
*GLCM_IMC1*	Second-order	–	–	0.0079	–
*GLCM_IMC2*	Second-order	–	–	0.0334	–
*GLCM_Cluster Shade*	Second-order	–	–	0.0166	–
*GLCM_Inverse Variance*	Second-order	–	–	0.0460	–
*GLCM_Maximum Probability*	Second-order	–	–	0.0004	–
*GLSZM_LAE*	High-order	0.0249	–	–	0.0109
*GLSZM_LAHGLE*	High-order	–	–	0.0048	–
*GLSZM_LALGLE*	High-order	–	–	–	0.0025
*GLRLM_SRE*	High-order	0.0311		–	–
*GLDM_DE*	Second-order	–	–	0.0086	–
*GLDM_LDLGLE*	Second-order	–	–	0.0001	0.0047
*NGTDM_Strength*	High-order	–	–	0.0009	–
Diffusion biomarkers					
*ADC_mean*	–	0.0292	–	–	–

ADC = apparent diffusion coefficient; CZ = Central Zone; DE = dependence entropy; GLCM = Gray-Level Co-Occurrence Matrix; GLDM = Gray Level Dependence Matrix; GLRLM = Gray Level Run Length Matrix; GLSZM = Gray Level Size Zone; IMC = informational measure of correlation; LAE = Large Area Emphasis; LAHGLE = Large Area Low Gray Level Emphasis; LDLGLE = Large Dependence Low Gray Level Emphasis; NGTDM = Neighborhood Gray Tone Difference Matrix; PZ = Peripheral Zone; SRE = short run emphasis; TZ = Transitional Zone; 2D = 2-dimensional; 3D = 3-dimensional.

**Table 3:** Logistic regression model performance.

	Performance	Sen.		Spe.		Acc.		AUC		*p*-Value	
Predictive Models											
		I	I + C	I	I + C	I	R + C	I	I + C	I	I + C
Risk groups											
CZ + TZ		0.408	0.542	0.690	0.604	0.561	0.574	0.610	0.651	0.603	0.318
PZ		0.667	0.563	0.631	0.717	0.648	0.644	0.670	0.685	**0.025**	0.190
Seminal vesicles		0.673	0.708	0.741	0.736	0.710	0.723	0.797	0.784	**0.005**	**0.035**
Whole prostate		0.551	0.604	0.724	0.679	0.645	0.644	0.659	0.693	0.524	0.386
BCR											
CZ + TZ		1.00	0.961	0.00	0.176	0.813	0.850	0.644	0.841	0.518	**0.020**
PZ		0.977	0.976	0.150	0.294	0.822	0.860	0.748	0.877	**0.025**	**0.002**
Seminal vesicles		0.977	0.976	0.250	0.412	0.841	0.880	0.788	0.862	0.104	0.080
Whole prostate		0.989	0.976	0.150	0.353	0.832	0.870	0.771	0.855	0.158	0.093
BCR (high/unf. IR)											
CZ + TZ		1.00	0.905	0.00	0.400	0.776	0.808	0.699	0.912	0.381	**0.032**
PZ		0.978	0.980	0.308	0.600	0.828	0.915	0.716	0.951	0.097	**0.001**
Seminal vesicles		0.981	0.976	0.308	0.600	0.846	0.904	0.756	0.898	0.246	**0.042**
Whole prostate		0.978	0.980	0.231	0.600	0.810	0.915	0.725	0.920	0.447	**0.017**

Acc. = accuracy; AUC = area under the curve; BCR = biochemical recurrence; CZ = Central Zone; I = imaging model; IR = intermediate risk; PZ = Peripheral Zone; TZ = Transitional Zone; R + C = radiomic + clinical model; Spe. = specificity; Sen. = sensitivity; unf. = unfavorable.

## Data Availability

The data presented in this study are available on request from the corresponding author.

## Supplementary Materials

The following supporting information can be downloaded at: https://www.mdpi.com/article/10.3390/cancers15164163/s1, Figure S1: A. Segmentation masks for the TZ + CZ, PZ and seminal vesicles in red, green and blue labels respectively, overlapped in the T2w sequence (high/unfavorable-intermediate risk patient experiencing BCR 10 years after diagnosis); B. ADC map from DWI (left) and K^trans^, k_ep_ and v_e_ maps (left) from DCE (high/unfavorable-intermediate risk patient with no BCR 10 years after diagnosis). Figure S2: Box plots for imaging (radiomics) biomarkers in each prostate region for biochemical recurrence 10 years after diagnosis in the central and transitional zones (A) and peripheral zone (B) (arbitrary units). ADC = apparent diffusion coefficient; GLRLM = Gray Level Run Length Matrix; GLSZM = Gray Level Size Zone; LAE = large area emphasis; SRE = short run emphasis. Figure S3: Box plots for imaging biomarkers in each prostate region for biochemical recurrence 10 years after diagnosis in the seminal vesicles (arbitrary units). GLCM = Gray-Level Co-Occurrence Matrix; GLDM = Gray Level Dependence Matrix; GLRLM = Gray Level Run Length Matrix; GLSZM = Gray Level Size Zone; IMC = informational measure of correlation; LAHGLE = Large Area Low Gray Level Emphasis; LDLGLE = Large Dependence Low Gray Level Emphasis; NGTDM = Neighborhood gray tone difference matrix. Figure S4: Box plots for imaging (radiomics) biomarkers in each prostate region for biochemical recurrence 10 years after diagnosis in the whole prostate gland (arbitrary units). GLCM = Gray-Level Co-Occurrence Matrix; GLDM = Gray Level Dependence Matrix; GLSZM = Gray Level Size Zone; ID = inverse difference; LAE = large area emphasis; LDLGLE = Large Dependence Low Gray Level Emphasis. Figure S5: Box plots for imaging biomarkers in each prostate region for biochemical recurrence 10 years after diagnosis in high/unfavorable-intermediate risk patients in the central and transitional zones (A) and peripheral zone (B) (arbitrary units). ADC = apparent diffusion coefficient; GLDM = Gray Level Dependence Matrix; GLSZM = Gray Level Size Zone; LAE = large area emphasis; LDE = large dependence emphasis; SDGHLE = Small Dependence High Gray Level Emphasis. Figure S6: Box plots for imaging biomarkers in each prostate region for biochemical recurrence 10 years after diagnosis in high/unfavorable-intermediate risk patients in the seminal vesicles (arbitrary units). ADC = apparent diffusion coefficient; GLCM = Gray-Level Co-Occurrence Matrix; GLDM = Gray Level Dependence Matrix; GLSZM = Gray Level Size Zone; IMC 1 = informational measure of correlation 1; LAHGLE = Large Area Low Gray Level Emphasis; LDLGLE = Large Dependence Low Gray Level Emphasis; p75 = percentile 75. Figure S7: Box plots for imaging biomarkers in each prostate region for biochemical recurrence 10 years after diagnosis in high/unfavorable-intermediate risk patients in the whole prostate gland (arbitrary units). ADC = apparent diffusion coefficient; GLCM = Gray-Level Co-Occurrence Matrix; GLDM = Gray Level Dependence Matrix; GLSZM = Gray Level Size Zone; LAE = large area emphasis; IV = inverse variance; LDE = large dependence emphasis; LDLGLE = Large Dependence Low Gray Level Emphasis; SZNUN = Size Zone Non Uniformity Normalized. Table S1: Patient distribution according to the available MRI sequences and their stratification risk. Table S2: Patient distribution according to the available MRI sequences and the presence or absence of biochemical recurrence 10 years from diagnosis. Table S3: Patient distribution according to the available MRI sequences and the presence or absence of biochemical recurrence 10 years from diagnosis in high/unfavorable-intermediate risk patients.
