# Supplementary material for: Prostate Region-Wise Imaging Biomarker Profiles for Risk Stratification and Biochemical Recurrence Prediction
Source: Cancers (Basel). 2023 Aug 18;15(16):4163. doi: 10.3390/cancers15164163 (PMC10453281; doi:10.3390/cancers15164163)
Supplement: Supplementary file 1 [file cancers-15-04163-s001.zip › Supplementary Figures.pdf]

Supplementary Figures

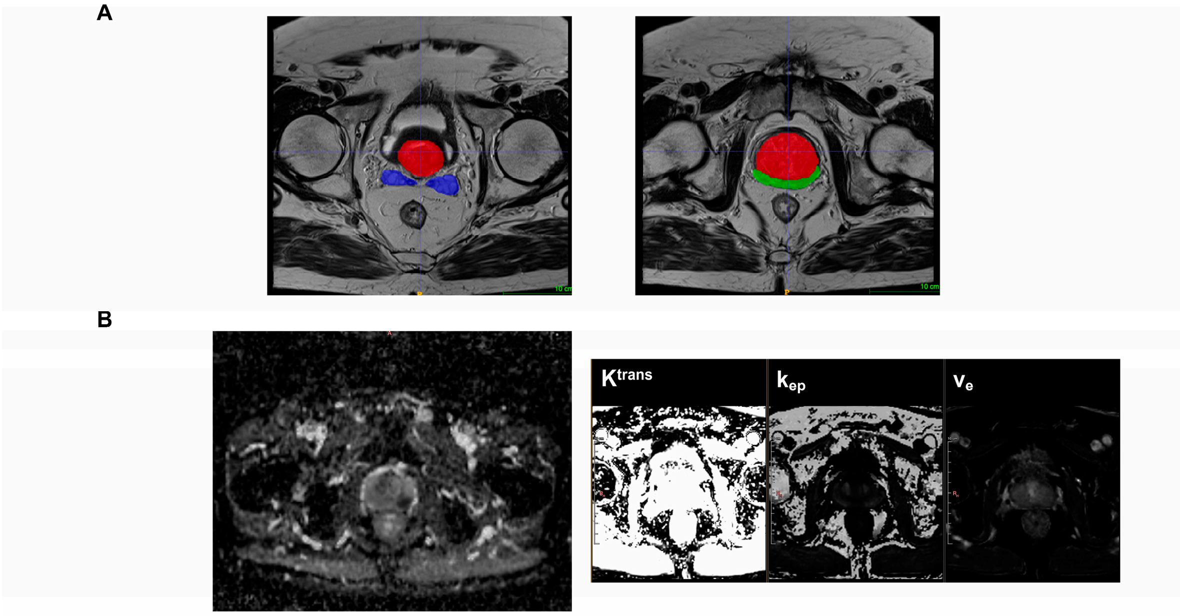

Figure S1: A. Segmentation masks for the TZ + CZ, PZ and seminal vesicles in red, green and blue labels respectively, overlapped in the T2w sequence (high/unfavorable-intermediate risk patient experiencing BCR 10 years after diagnosis); B. ADC map from DWI (left) and K^{trans}, k_{ep} and v_e maps (left) from DCE (high/unfavorable-intermediate risk patient with no BCR 10 years after diagnosis).

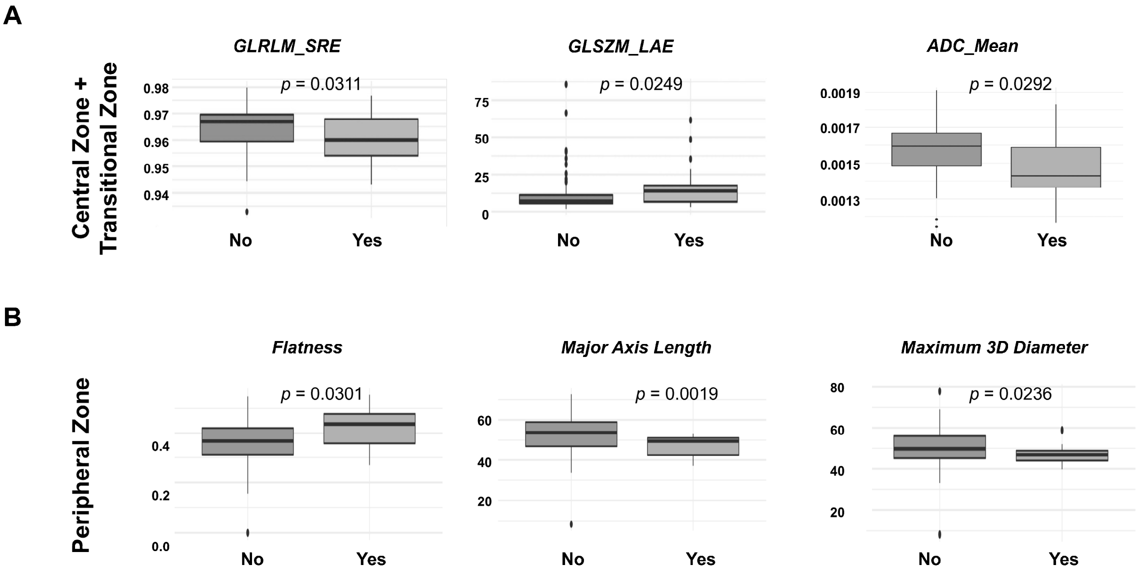

Figure S2: Box plots for imaging (radiomics) biomarkers in each prostate region for biochemical recurrence 10 years after diagnosis in the central and transitional zones (A) and peripheral zone (B) (arbitrary units). ADC = apparent diffusion coefficient; GLRLM = Gray Level Run Length Matrix; GLSZM = Gray Level Size Zone; LAE = large area emphasis; SRE = short run emphasis.

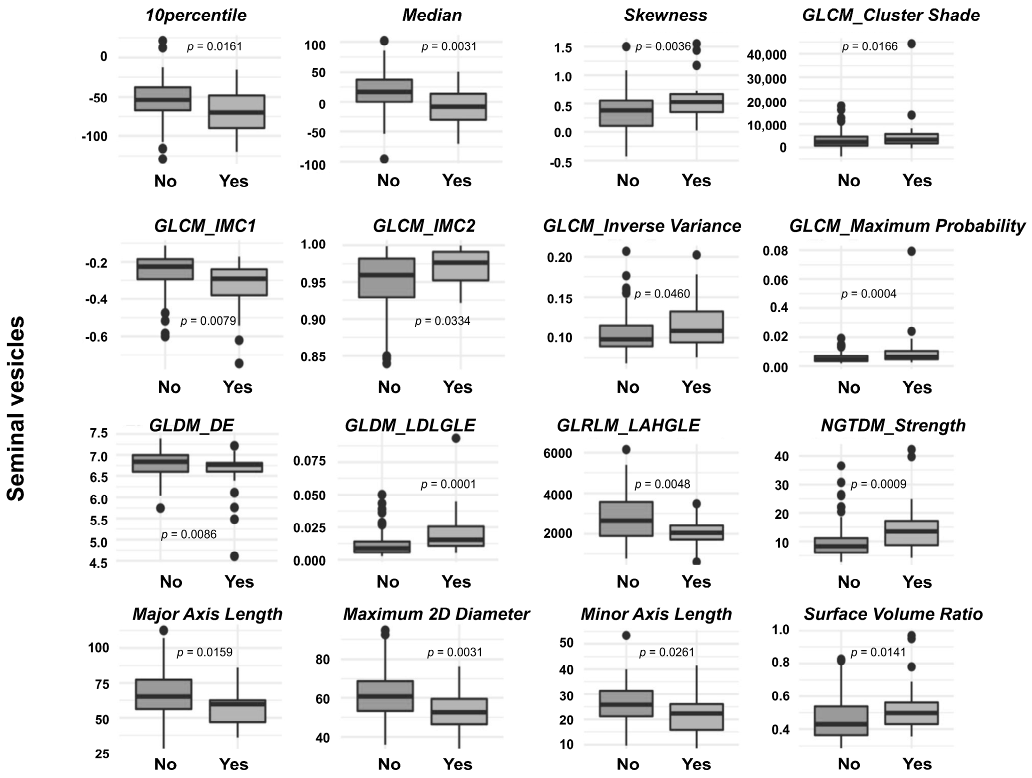

Figure S3: Box plots for imaging biomarkers in each prostate region for biochemical recurrence 10 years after diagnosis in the seminal vesicles (arbitrary units). GLCM = Gray-Level Co-Occurrence Matrix; GLDM = Gray Level Dependence Matrix; GLRLM = Gray Level Run Length Matrix; GLSZM = Gray Level Size Zone; IMC = informational measure of correlation; LAHGLE = Large Area Low Gray Level Emphasis; LDLGLE = Large Dependence Low Gray Level Emphasis; NGTDM = Neighborhood gray tone difference matrix.

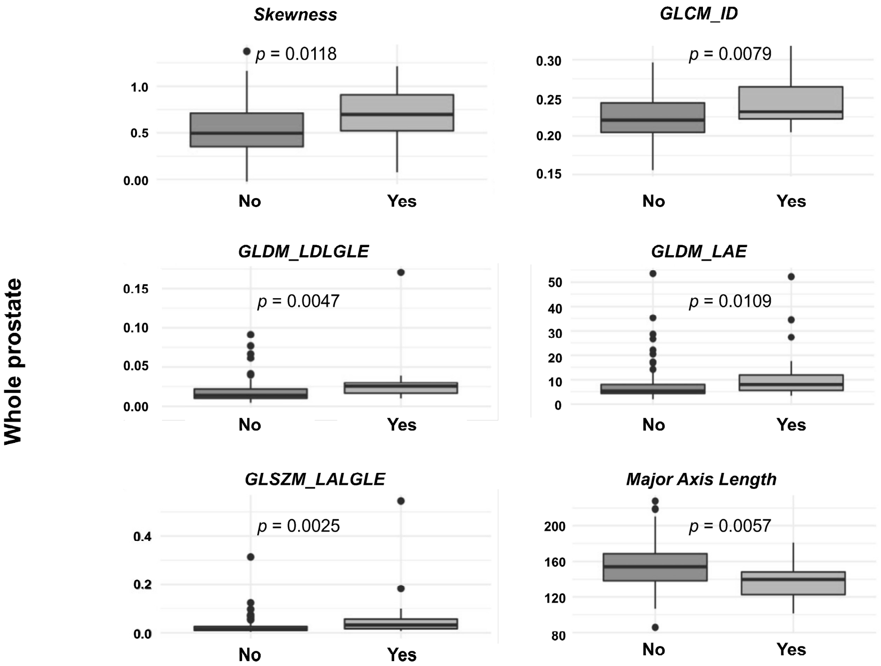

Figure S4: Box plots for imaging (radiomics) biomarkers in each prostate region for biochemical recurrence 10 years after diagnosis in the whole prostate gland (arbitrary units). GLCM = Gray-Level Co-Occurrence Matrix; GLDM = Gray Level Dependence Matrix; GLSZM = Gray Level Size Zone; ID = inverse difference; LAE = large area emphasis; LDLGLE = Large Dependence Low Gray Level Emphasis.

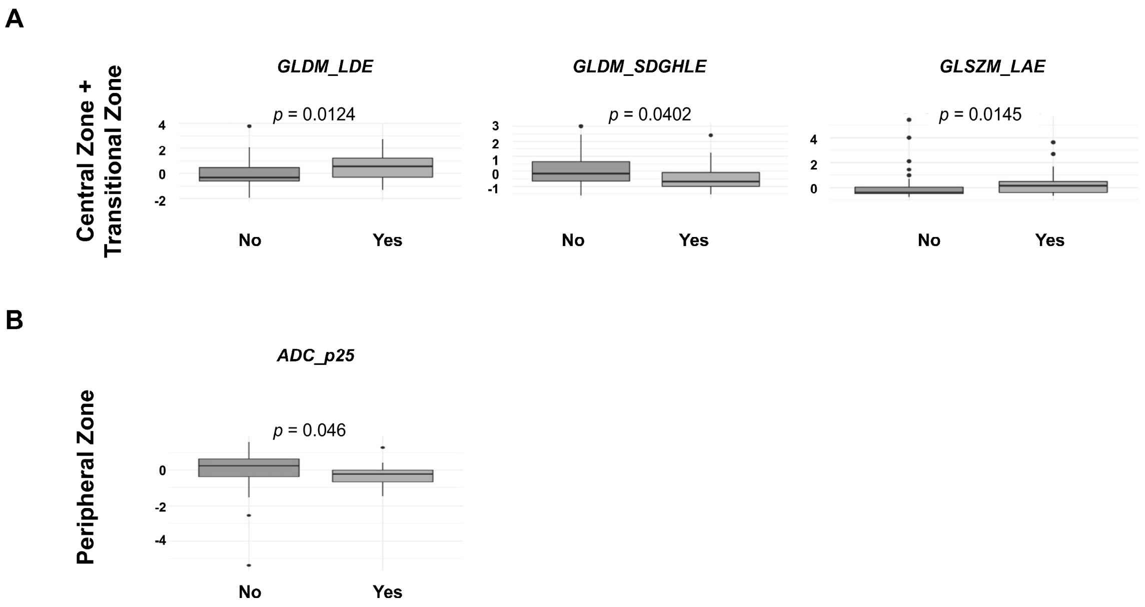

Figure S5: Box plots for imaging biomarkers in each prostate region for biochemical recurrence 10 years after diagnosis in high/unfavorable-intermediate risk patients in the central and transitional zones (A) and peripheral zone (B) (arbitrary units). ADC = apparent diffusion coefficient; GLDM = Gray Level Dependence Matrix; GLSZM = Gray Level Size Zone; LAE = large area emphasis; LDE = large dependence emphasis; SDGHLE = Small Dependence High Gray Level Emphasis.

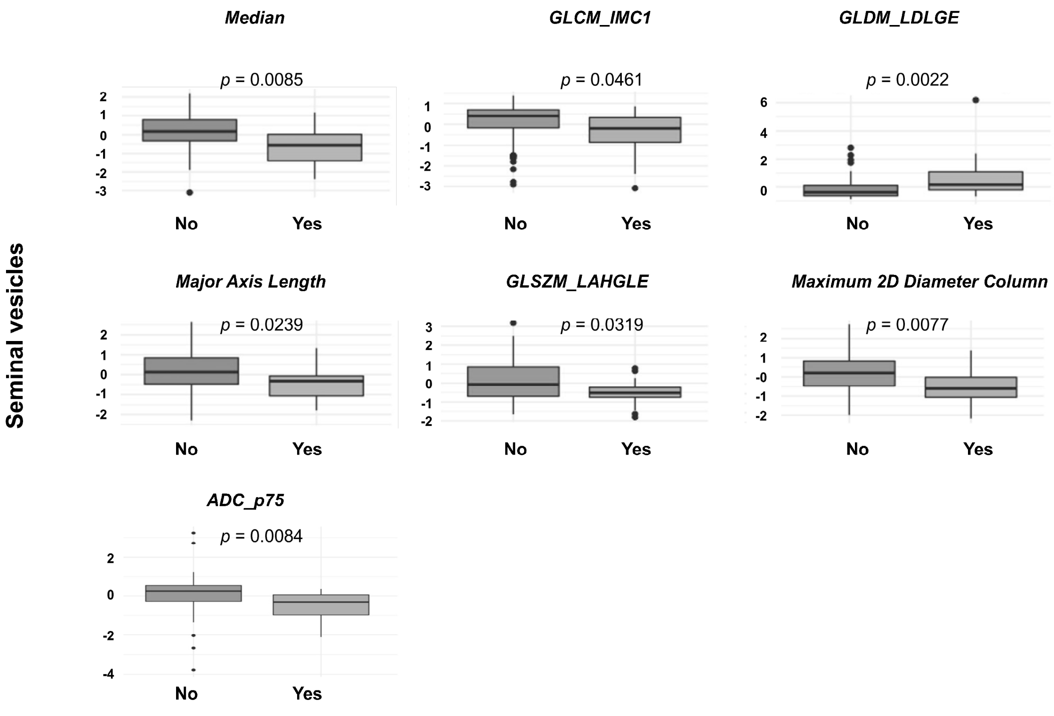

Figure S6: Box plots for imaging biomarkers in each prostate region for biochemical recurrence 10 years after diagnosis in high/unfavorable-intermediate risk patients in the seminal vesicles (arbitrary units). ADC = apparent diffusion coefficient; GLCM = Gray-Level Co-Occurrence Matrix; GLDM = Gray Level Dependence Matrix; GLSZM = Gray Level Size Zone; IMC 1 = informational measure of correlation 1; LAHGLE = Large Area Low Gray Level Emphasis; LDLGLE = Large Dependence Low Gray Level Emphasis; p75 = percentile 75.

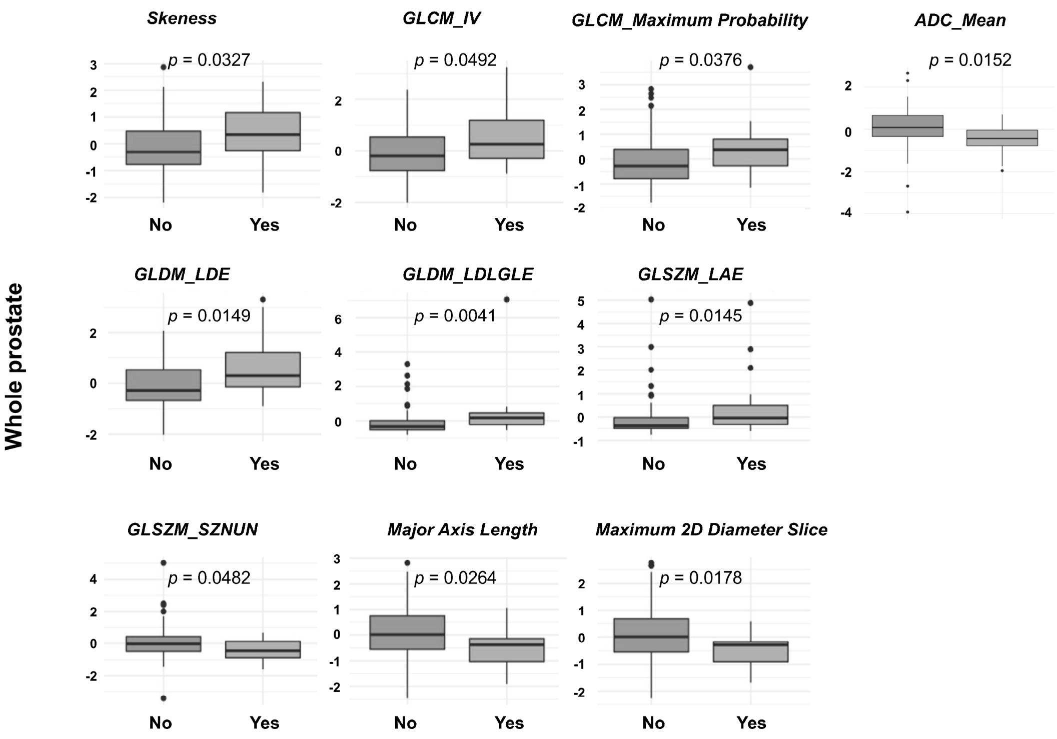

Figure S7: Box plots for imaging biomarkers in each prostate region for biochemical recurrence 10 years after diagnosis in high/unfavorable-intermediate risk patients in the whole prostate gland (arbitrary units). ADC = apparent diffusion coefficient; GLCM = Gray-Level Co-Occurrence Matrix; GLDM = Gray Level Dependence Matrix; GLSZM = Gray Level Size Zone; LAE = large area emphasis; IV = inverse variance; LDE = large dependence emphasis; LDLGLE = Large Dependence Low Gray Level Emphasis; SZNUN = Size Zone Non Uniformity Normalized.
