# Supplementary material for: Prostate Region-Wise Imaging Biomarker Profiles for Risk Stratification and Biochemical Recurrence Prediction
Source: Cancers (Basel). 2023 Aug 18;15(16):4163. doi: 10.3390/cancers15164163 (PMC10453281; doi:10.3390/cancers15164163)
Supplement: Supplementary file 1 [file cancers-15-04163-s001.zip › Supplementary Tables.pdf]

Supplementary Tables

Table S1. Patient distribution according to the available MRI sequences and their stratification risk.

Analysis	Sample size	Low/favorable-IR	High/unfavorable-IR
Texture	128	32	96
Diffusion	107	31	76
Perfusion	62	24	38

IR = intermediate risk.

Table S2. Patient distribution according to the available MRI sequences and the presence or absence of biochemical recurrence 10 years from diagnosis.

Analysis	Sample size	No BCR	BCR
Texture	128	108	20
Diffusion	107	92	15
Perfusion	62	54	8

BCR = biochemical recurrence.

Table S3. Patient distribution according to the available MRI sequences and the presence or absence of biochemical recurrence 10 years from diagnosis in high/unfavorable-intermediate risk patients.

Analysis	Sample size	No BCR	BCR
Texture	96	78	18
Diffusion	76	63	13
Perfusion	38	32	6

BCR = biochemical recurrence.
